# Methylation of ZNF331 is an independent prognostic marker of colorectal cancer and promotes colorectal cancer growth

**DOI:** 10.1186/s13148-017-0417-4

**Published:** 2017-10-18

**Authors:** Yuzhu Wang, Tao He, James G. Herman, Enqiang Linghu, Yunsheng Yang, François Fuks, Fuyou Zhou, Linjie Song, Mingzhou Guo

**Affiliations:** 1 Department of Gastroenterology & Hepatology, Chinese PLA General Hospital, 28 Fuxing Road, Beijing, 100853 China; 2 Department of Geriatric Digestive System, Chinese PLA Navy General Hospital, 6 Fucheng Road, Beijing, 100048 China; 3 The Hillman Cancer Center, University of Pittsburgh Cancer Institute, Pittsburgh, PA 15213 USA; 4 Laboratory of Cancer Epigenetics, Free University of Brussels (U.L.B.), 1070 Brussels, Belgium; 5 Department of Thoracic Surgery, Anyang Tumor Hospital, Anyang, 455000 China; 6 Department of General Surgery, Chinese PLA General Hospital, 28 Fuxing Road, Beijing, 100853 China; 7 Medical College of NanKai University, Tianjin, 300071 China

**Keywords:** ZNF331, Epigenetics, DNA methylation, Colorectal cancer

## Abstract

**Background:**

ZNF331 was reported to be a transcriptional repressor. Methylation of the promoter region of ZNF331 has been found frequently in human esophageal and gastric cancers. The function and methylation status of ZNF331 remain to be elucidated in human colorectal cancer (CRC).

**Methods:**

Six colorectal cancer cell lines, 146 cases of primary colorectal cancer samples, and 10 cases of noncancerous colorectal mucosa were analyzed in this study using the following techniques: methylation specific PCR (MSP), qRT-PCR, siRNA, flow cytometry, xenograft mice, MTT, colony formation, and transfection assays.

**Results:**

Loss of ZNF331 expression was found in DLD1 and SW48 cells, reduced expression was found in SW480, SW620, and HCT116 cells, and high level expression was detected in DKO cells. Complete methylation of the ZNF331 in the promoter region was found in DLD1 and SW48 cells, partial methylation was found in SW480, SW620, and HCT116 cells, and unmethylation was detected in DKO cells. Loss of/reduced expression of ZNF331 is correlated with promoter region methylation. Restoration of ZNF331 expression was induced by 5-aza-2′-deoxycytidine (DAC) in DLD1 and SW48 cells. These results suggest that ZNF331 expression is regulated by promoter region methylation in CRC cells. ZNF331 was methylated in 67.1% (98/146) of human primary colorectal cancer samples. Methylation of ZNF331 was significantly associated with tumor size, overall survival (OS), and disease-free survival (DFS) (*p* < 0.01, *p* < 0.01, *p* < 0.05). Methylation of ZNF331 was an independent poor prognostic marker for 5-year OS and 5-year DFS (both *p* < 0.05). ZNF331 suppressed cell proliferation and colony formation in CRC cells and suppressed human CRC cell xenograft growth in mice.

**Conclusions:**

ZNF331 is frequently methylated in human colorectal cancer, and the expression of ZNF331 is regulated by promoter region methylation. Methylation of ZNF331 is a poor prognostic marker of CRC.

## Background

Colorectal cancer (CRC) is the third most commonly diagnosed cancer in males and the second in females worldwide [1]. The incidence has sharply increased in the past two decades in Eastern countries with changing environmental factors, such as lifestyle and diet [2, 3]. Accumulation of aberrant genetic and epigenetic changes plays an important role in CRC initiation and progression [4]. Numerous prospective cohort epidemiology studies have identified specific dietary and lifestyle factors that either promote or protect against CRC [5–7]. Consumption of red meat and animal fats increases CRC risk, whereas dietary fiber decreases risk [8, 9]. Other studies suggest that the human gut microbiome is relatively stable in individuals over time except in the case of certain events such as food poisoning/infection or international travel. This likely reflects the hegemony of long-term dietary patterns on our gut microbiome [10, 11]. All these factors may cause colorectal epithelial cell epigenetic changes and further induces tumorigenesis. Thus, identification of new epigenetic biomarkers for diagnosis, prognosis, prediction, and targeting therapy for CRC is necessary.

Zinc finger protein 331 (ZNF331) was first identified from thyroid tumors [12]. It is also known as RITA (rearranged in thyroid adenoma), ZNF361, and ZNF463 [13]. The ZNF331 gene is located at chromosome 19q13.42, a region in which loss of heterozygosity (LOH) was detected in prostate cancer [14]. In our previous study, we found that the ZNF331 gene is frequently methylated in human esophageal squamous cell cancer (ESCC) and it serves as a tumor suppressor in ESCC [15]. The function of ZNF331 in human CRC remains unclear. In this study, we analyzed the epigenetic regulation and the function of ZNF331 in human CRC.

## Methods

### Human tissue samples and cell lines

A total of 146 cases of primary CRC and 10 cases of noncancerous colorectal mucosa were collected from the Chinese PLA General Hospital in Beijing between May 2009 and November 2013. All cancer samples were classified according to WHO Classification of Digestive Tumors: the 4th Edition. All samples were collected under the guidelines approved by the institutional review board at the Chinese PLA General Hospital. Among the patient cases, 98 cases were male and 48 cases were female. The median age was 60 years old (range 33–86 years old). All cancer samples were classified according to the TNM staging system (AJCC2010), which included tumor stage I (*n* = 17), stage II (*n* = 58), stage III (*n* = 52), and stage IV (*n* = 19). Six CRC cell lines, DLD1, SW48, HCT116, SW480, SW620, and DKO (DNMT1 and DNMT3b double knockout from HCT116 cells, a generous gift from Stephen Baylin), were examined in this study and maintained in 90% Roswell Park Memorial Institute (RPMI) 1640 media supplemented with 10% fetal bovine serum, 100 U/ml penicillin and 100 mg/ml streptomycin. HEK-293T cells were cultured in Dulbecco’s modified Eagle’s medium supplemented with 10% fetal bovine serum. All cell lines were cultured in an atmosphere of 5% carbon dioxide at 37 °C.

### 5-Aza-2′-deoxycytidine treatment

CRC cell lines (DLD1, SW48, HCT116, SW480, SW620, and DKO) were split to a low density (30% confluence) 12 h before treatment with 2 μM 5-aza-2′-deoxycytidine (DAC, Sigma, MO, USA). Growth medium conditioned with DAC at 2 μM was exchanged every 24 h for a total of 96 h. At the end of the treatment course, RNA was extracted from the cells as described below.

### RNA isolation, semi-quantitative RT-PCR, and real-time quantitative RT-PCR analyses

Total RNA was isolated by Trizol reagent (Life Technologies, MD, USA). Agarose gel electrophoresis and spectrophotometric analysis were used to check RNA quality and quantity. Total RNA (5 μg) was used to synthesize first-strand complementary DNA (cDNA) according to the manufacturer’s instructions (Invitrogen, Carlsbad, CA). The reaction mixture was diluted to 100 μl with water, and 2.5 μl of diluted cDNA mixture was added to each 25 μl PCR reaction. The ZNF331 PCR primer sequences were as follows: 5′-TAGGTCAGCTCTAGCCTCTC-3′ (forward) and 5′-AGCGTACCTTCACATATCCAG-3′ (reverse). Thermal cycling parameters were as follows: 95 °C 5 min; (95 °C 30 s, 58 °C 30 s, and 72 °C 30 s) 35 cycles; 72 °C 5 min. The primers for GAPDH were as follows: 5′-GAGTCAACGGATTTGGTCGT-3′ (forward), and 5′-GACAAGCTTCCCGTTCTCAG-3′ (reverse). Thermal cycling parameters were as follows: 95 °C 5 min; (95 °C 30 s, 58 °C 30 s and 72 °C 40 s) 25 cycles; 72 °C 5 min. The amplified PCR products were examined by 1.5% agarose gels. Each cDNA sample was analyzed in triplicate with the Applied Biosystems StepOnePlus Real-Time PCR Systems using SYBR Green Realtime PCR Master Mix (Toyobo, Shanghai, China) according to the manufacturer’s instructions. The relative amount of ZNF331 mRNA was normalized to GAPDH using the ΔΔCt method.

### Bisulfite modification, methylation-specific PCR, bisulfite sequencing, and KRAS and BRAF mutation detection

Genomic DNA was extracted by the proteinase K method. Cultured cells and fresh tissue samples were digested by DNA digestion buffer (pH 8.0, 10 mM Tris-Cl, 25 mM EDTA, 1% SDS, 100 μg/ml proteinase K) and extracted by phenol/chloroform. The bisulfite modification assay was performed as previously described [16]. MSP primers were designed according to genomic sequences around the transcription start sites (TSS) and synthesized (BGI, Beijing, China) to detect unmethylated (U) and methylated (M) alleles. The MSP primers were as follows: 5′-TAAGGTAGGACGTTTTTAGGGTCGC-3′ (MF) and 5′-AACTCTACACGACGCAAATAAAACCG-3′ (MR); 5′-TTTTAAGGTAGGATGTTTTTAGGGTTGT-3′ (UF) and 5′-ACAACTCTACACAACACAAATAAAACCA-3′ (UR). The thermal cycling parameters were as follows: 95 °C 5 min; (95 °C 30 s, 60 °C 30 s and 72 °C 40 s) 35 cycles; 72 °C 5 min. The expected sizes of unmethylated and methylated products were 147 and 142 bp, respectively. Bisulfite-treated DNA was also amplified using bisulfite sequencing (BSSQ) primers that included the MSP region. The sequencing primers were as follows: 5′-GGTTATGAGTTATATTTTTTAGAAG-3′ (forward) and 5′-CTCRCTCCTCATTAAACTATAC-3′ (reverse). The thermal cycling parameters were as follows: 95 °C 5 min; (95 °C 30 s, 55 °C 30 s and 72 °C 40 s) 35 cycles; 72 °C 5 min. Methylation status was detected by MSP in four genes (*RUNX3*, *CACNA1G*, *IGF2*, and *MLH1*) to represent CpG island methylator phenotype (CIMP) according to a report from Ogino et al. [17], CIMP-high was defined as ≥ 3 of 4 markers methylated. The primer sequences are listed in Table 1. KRAS codons 12 and 13 and BRAF codon 600 were amplified by PCR and sequenced according to previous reports [18, 19]. The primer sequences are listed in Table 1.

**Table 1:** Primer sequences and PCR conditions

Gene	Forward primer	Reverse primer	Amplicon size (bp)	Annealing temperature (°C)	PCR cycles
ZNF331 U	5′-TTTTAAGGTAGGATGTTTTTAGGGTTGT-3′	5′-ACAACTCTACACAACACAAATAAAACCA-3′	147	60	35
ZNF331 M	5′-TAAGGTAGGACGTTTTTAGGGTCGC-3′	5′-AACTCTACACGACGCAAATAAAACCG-3′	142	60	35
CANA1G U	5′-TTTTTTTGTTTTGTGTTTAGGTTTT-3′	5′-CCCTCTCAAAACAACTTCACCA-3′	71	64	35
CANA1G M	5′-TCGTTTCGCGTTTAGGTTTC-3′	5′-CTCGAAACGACTTCGCCG-3′	62	64	35
IGF2 U	5′-GGAGTGGTTTTGGTGTTGTTATT-3′	5′-CCCAACTCAATTTAAACCAACA-3′	90	66	35
IGF2 M	5′-GCGGTTTCGGTGTCGTTATC-3′	5′-CCAACTCGATTTAAACCGACG-3′	86	68	30
RUNX3 U	5′-GTTGGTGGATTATGTAGGTGAGTTT-3′	5′-ACTCACCTTAAAAACAACAAACAACA-3′	115	66	35
RUNX3 M	5′-GCGGATTACGTAGGCGAGTTC-3′	5′-ACCTTAAAAACGACGAACAACG-3′	107	66	35
MLH1 U	5′-TTTTGATGTAGATGTTTTATTAGGGTTGT-3′	5′-ACCACCTCATCATAACTACCCACA-3′	124	60	35
MLH1 M	5′-ACGTAGACGTTTTATTAGGGTCGC-3′	5′-CCTCATCGTAACTACCCGCG-3′	115	60	35
KRAS exon2	5′-AAGGTGAGTTTGTATTAAAAGGTACTGG-3′	5′-TGGTCCTGCACCAGTAATATGC-3′	264	58	35
BRAF- 600E	5′-TCATAATGCTTGCTCTGATAGGA-3′	5′-CTTTCTAGTAACTCAGCAGC-3′	251	60	35

### Immunohistochemistry

Immunohistochemistry (IHC) was performed in primary colorectal cancer samples and matched adjacent tissue samples. The ZNF331 antibody (Biosynthesis Biotechnology, Beijing, China) was diluted 1:50. The staining intensity and extent of the staining area were scored using the German semi-quantitative scoring system. The staining intensity of expression was quantified as follows: no staining = 0; weak staining = 1; moderate staining = 2; and strong staining = 3. The extent of DACT2 expression was quantified as follows: 0% = 0, 1–24% = 1, 25–49% = 2, 50–74% = 3, and 75–100% = 4. The final immunoreactive score (0 to 12) was determined by multiplying the intensity score and the extent of stained cells score.

### Construction of lentiviral ZNF331 expression vectors and selection of stable expression cells

The human full-length ZNF331 cDNA (NM_001079906) was cloned into the pLenti6-3×FLAG vector. The primers were as follows: 5′-GGAAGATCTATGGCCCAGGGTTTGGTG-3′ (forward) and 5′-GGGCCCTCAACTGTTGTGGATCCTCTG-3′ (reverse). The HEK-293T cell line was maintained in 90% DMEM (Invitrogen, CA, USA) supplemented with 10% fetal bovine serum. ZNF331 expressing lentiviral vector was transfected into HEK-293T cells (5 × 10^6^ per 100 mm dish) using Lipofectamine 3000 Reagent (Invitrogen, CA, USA) at a ratio of 1:3 (DNA mass: Lipo mass). Viral supernatant was collected and filtered after 48 h. DLD1 and SW48 cells were then infected with viral supernatant. Cells stably expressing ZNF331 were selected with Blasticidin (Life Technologies, MD, USA) at concentrations of 0.5 μg/ml (DLD1) and 3 μg/ml (SW48) for 2 weeks.

### siRNA knockdown technique

Selected small interfering RNA (siRNAs) targeting ZNF331 and RNAi negative control duplex (Gene Pharma, Shanghai, China) were used in this study. The sequences were as follows: siRNA-2156 duplex, 5′-GACUACGAAUGCAAAGACUTT-3′ and 5′-AGUCUUUGCAUUCGUAGUCTT-3′; RNAi negative control duplex, 5′-UUCUCCGAACGUGUCACGUTT-3′ and 5′-ACGUGACACGUUCGGAGAATT-3′. RNAi oligonucleotide or RNAi negative control duplex was transfected into ZNF331 highly expressed DKO cells using Lipofectamine RNAiMAX (Invitrogen, CA, USA).

### Cell viability assay

ZNF331 stably expressed and unexpressed DLD1 and SW48 cells were plated into 96-well plates at a density of 2 × 10^3^ cells/well. The cells (5 × 10^3^) were plated into 96-well plates before and after knockdown of ZNF331 in DKO cells. The cell viability was measured by the MTT assay at 0, 24, 48, and 72 h (KeyGEN Biotech, Nanjing, China). Absorbance was measured on a microplate reader (Thermo Multiskan MK3, MA, USA) at a wave length of 492 nm. The results were plotted as means ± SD.

### Colony formation assay

ZNF331 stably expressed and unexpressed DLD1 and SW48 cell lines were seeded in six-well plates at a density of 200 cells per well. DKO cells before and after knockdown of ZNF331 were seeded in six-well plates at a density of 200 cells per well. After 2 weeks, the cells were fixed with 75% ethanol for 30 min. Colonies were then stained with 0.5% crystal violet solution and counted. Each experiment was repeated three times.

### Flow cytometry for cell cycle

ZNF331 stably expressed and unexpressed DLD1 and SW48 cell lines were plated into six-well plates at 1 × 10^6^ cells per well. The cells were harvested at 48 h, washed with phosphate-buffered saline twice, and stained according to the manufacturer’s instructions for the Cycletest™ Plus DNA Reagent Kit (Becton, Dickinson and Company, CA, USA). The samples were analyzed with a FACS Caliber flow cytometer (Becton, Dickinson and Company). Each experiment was repeated three times.

### Protein preparation and Western blotting

The cells were lysed in ice-cold Tris buffer (20 mmol/l Tris; pH 7.5) containing 137 mmol/l NaCl, 2 mmol/l ethylenediaminetetraacetic acid, 1% Triton X, 10% glycerol, 50 mmol/l NaF, 1 mmol/l DTT, and a protease inhibitor cocktail (Roche Applied Science, IN, USA). Protein concentrations were quantified using the BCA protein assay kit (CWBIO, Beijing, China). The protein lysates were then separated by SDS-PAGE and electroblotted onto polyvinylidene fluoride (PVDF) membranes (Hybond-P). After blocking with 5% nonfat milk and 0.1% Tween-20 in Tris-buffered saline (TBS), the membranes were incubated with antibodies. The antibodies for immunoblot analysis were as follows: FLAG-tag (1:2000, Proteintech, IL, USA), cyclin D1 (1:500, Proteintech, IL, USA), ZNF331(1:500 Biosynthesis Biotechnology, Beijing, China), cyclin E1 (1:500, Proteintech, IL, USA), and GAPDH (1:2000, Proteintech, IL, USA). GAPDH antibody was used as a loading control. The blots were visualized using enhanced chemiluminescence (PierceBioscience, IL, USA).

### ZNF331 unexpressed and re-expressed DLD1 cell xenograft mouse model

Stably transfected DLD1 cell line with pLenti6 vector or pLenti6-ZNF331 vector (4 × 10^6^ cells in 0.15 ml phosphate-buffered saline) were injected subcutaneously into the dorsal right side of 4-week-old male Balb/c nude mice. Each group included five mice. Tumor volumes were measured every 4 days starting 6 days after implantation. Tumor volumes were calculated according to the formula: *V* = *L* × *W* ^2^/2, where *V* represents volume (mm^3^), *L* represents biggest diameter (mm), and *W* represents smallest diameter (mm). The mice were sacrificed on the 22nd day, and tumor weights were measured. All procedures were approved by the Animal Ethics Committee of the Chinese PLA General Hospital.

### Statistical analysis

Statistical analysis was performed using SPSS 18.0 software (SPSS, IL, USA). Either *χ* ^2^ or Fisher’s exact tests were used to evaluate the relationship between methylation status and clinicopathological characteristics. The two-tailed independent samples’ *t* test was applied to determine the statistical significance of the differences between the two experimental groups. Survival rates were calculated by the Kaplan–Meier method, and differences in survival curves were evaluated using the log-rank test. Cox proportional hazards models were fit to determine independent associations of ZNF331 methylation with overall survival (OS) and disease-free survival (DFS) outcomes. Two-sided tests were used to determine significance, and *p* < 0.05 was considered statistically significant.

## Results

### The expression of ZNF331 is silenced by promoter region hypermethylation in human CRC cell lines

**Fig. 1:**
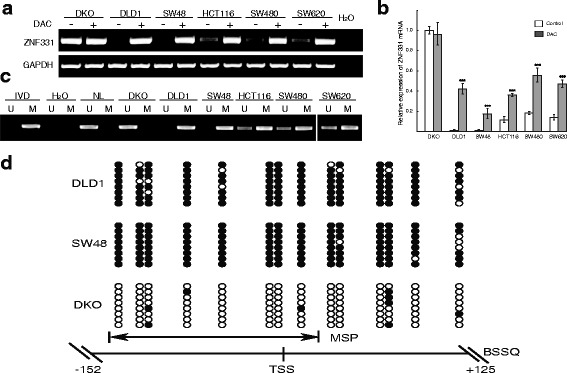
Expression and methylation status of ZNF331 in CRC cells. **a** Expression of ZNF331 was detected by semi-quantitative RT-PCR in CRC cells. H_2_O: double-distilled water, negative control. GAPDH: internal control. DAC 5-aza-2′-dexycytidine, (−) absence of DAC, (+) presence of DAC. **b** Relative mRNA expression level of ZNF331 before and after treatment with DAC was detected by real-time quantitative RT-PCR. (***p < 0.01). **c** MSP results in CRC cell lines. IVD in vitro methylated DNA (methylation control), NL lymphocyte DNA (unmethylation control), U unmethylated alleles, M methylated alleles. **d** Bisulfite sequencing results: double-headed *arrow* indicates the region of the MSP product. Filled circles: methylated CpG sites; *open circles*: unmethylated CpG sites. TSS: transcription start site

The expression of ZNF331 was examined by semi-quantitative reverse transcription PCR (RT-PCR) and real-time quantitative RT-PCR in CRC cells. ZNF331 was highly expressed in DKO cells. Loss of ZNF331 expression was found in DLD1 and SW48 cells, and reduced expression was found in HCT116, SW480, and SW620 cells (Fig. 1a, b, all *p* < 0.01). Methylation of the ZNF331 promoter region was detected by methylation-specific PCR (MSP). Complete methylation was observed in DLD1 and SW48 cells, partial methylation was detected in HCT116, SW480, and SW620 cells, and unmethylation was found in DKO cells (Fig. 1c). These data indicate that loss of/reduced expression of ZNF331 is correlated with promoter region methylation in CRC cell lines. To further determine whether expression of ZNF331 was regulated by promoter region methylation, CRC cells were treated with 5-aza-2′-deoxycytidine (DAC). As expected, restoration of ZNF331 expression was found in DLD1 and SW48 cells after DAC treatment, and increased expression was detected in HCT116, SW480, and SW620 cells (Fig. 1a, b, all *p* < 0.01). These results suggested that ZNF331 expression is regulated by promoter region methylation in human CRC cell lines. To validate the MSP results, bisulfite sequencing (BSSQ) was employed. Dense methylation was observed in the promoter region of ZNF331 in DLD1 and SW48 cells, while unmethylation was found in DKO cells (Fig. 1d).

### ZNF331 is frequently methylated in human primary CRC, and methylation of ZNF331 is a poor prognostic marker

**Fig. 2:**
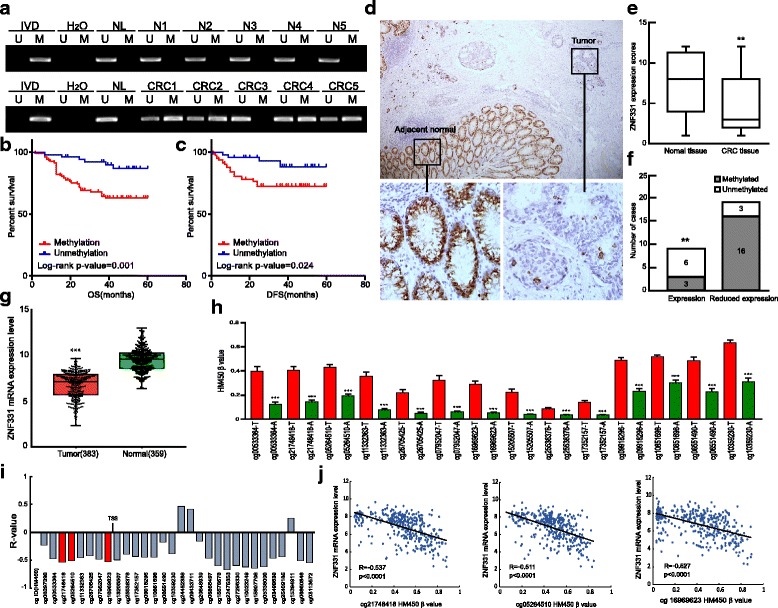
Expression and methylation status of ZNF331 in primary CRC. **a** Representative results of MSP for ZNF331 in primary CRC samples and matched adjacent tissue samples. N normal colorectal mucosa. **b**, **c** Kaplan–Meier curves show the association between ZNF331 methylation and OS and DFS in CRC patients. *Blue*: ZNF331 unmethylated CRC patients (*n* = 48); *red*: ZNF331 methylated CRC patients (*n* = 98) (*p* = 0.001, *p* = 0.024, log-rank test). **d** Representative IHC results show the expression of ZNF331 in CRC samples and matched adjacent tissue samples (upper, ×100; lower, ×400). **e** ZNF331 expression scores are shown as *box* plots, and the *horizontal lines* represent the median score; the bottom and top of the *boxes* represent the 25th and 75th percentiles, respectively; vertical bars represent the range of data (**p < 0.01). **f** The bar diagram shows the expression and DNA methylation status of ZNF331 in different cancer samples. Reduced expression of ZNF331 was significantly associated with promoter region methylation (**p < 0.01). **g** TCGA data and GTEx data show ZNF331 mRNA expression levels in CRC tissues (*n* = 383) and normal colorectal mucosa (*n* = 359) according to RNA-seq results. *Box* plots: the levels of ZNF331 expression. *Horizontal lines*: counts of log2 (TPM + 1). TPM Transcripts Per Million (reads) (***p < 0.001). **h** Methylation status of 14 CpG sites in promoter region of human primary CRC tissue samples (T, shown in *red*) (*n* = 356) compared to adjacent normal colorectal tissues (A, shown in *green*) (*n* = 21) (***p < 0.001). **i** The correlation between expression level of ZNF331 and methylation status of CpG sites in the vicinity of the TSS. **j** Methylation status of top three CpG sites in promoter region (shown in *red* in **i**) are correlated with loss of/reduced ZNF331 expression in 356 cases of CRC (all *p* < 0.0001)

Methylation of ZNF331 was detected by MSP in primary CRC samples and normal colorectal mucosa. ZNF331 was methylated in 67.1% (98/146) of primary CRC and 0% (0/10) of normal colorectal mucosa (Fig. 2a). Methylation of ZNF331 was significantly associated with tumor size (*p* < 0.05, Table 2), while no association was found between ZNF331 methylation and gender, age, tumor differentiation, lymphatic metastasis, tumor location, and TNM stages (all *p* > 0.05, Table 2). KRAS and BRAF mutation was detected in 30.15% (41/136) and 2.94% (4/136) of primary CRC. No association was found between ZNF331 methylation and KRAS or BRAF mutations (all *p* > 0.05, Table 3). CIMP-high was detected in 11.03% (15/136) of primary CRC. The methylation frequency of four markers (RUNX3, CACNA1G, IGF2, and MLH1) is 10.29% (14/136), 21.32% (29/136), 25% (34/136), and 15.44% (21/136). No association was found between ZNF331 methylation and CIMP-high or each marker (*p* > 0.05, Table 3). Kaplan–Meier plots indicated that methylation of ZNF331 was significantly associated with poor 5-year overall survival (OS) (*p* = 0.001, Fig. 2b) and 5-year disease-free survival (DFS) (*p* = 0.024, Fig. 2c). According to Cox proportional hazards model analysis, ZNF331 methylation was an independent prognostic factor for poor 5-year OS and 5-year DFS after adjusting for tumor differentiation and TNM stage (both *p* < 0.05, Table 4).

**Table 2:** Associations between clinicopathological features and ZNF331 methylation status in colorectal cancer patients

Clinical parameter	Number	ZNF331 methylation status		*p* value
		Methylated	Unmethylated	
Age(years)				
≥ 60	74	52	22	0.482
< 60	72	46	26	
Gender				
Male	98	67	31	0.709
Female	48	31	17	
Tumor differentiation				
Moderate and well	106	69	37	0.436
Poor	40	29	11	
TNM stage				
I–II	75	47	28	0.291
III–IV	71	51	20	
Tumor size				
< 5 cm	75	42	33	0.005**
≥ 5 cm	71	56	15	
Tumor location				
Right	54	41	13	0.101
Left	92	57	35	

Chi-square test; **p < 0.01

**Table 3:** KRAS and BRAF mutations, CpG island methylator phenotype (CIMP), and ZNF331 methylation status in colorectal cancer patients

Marker genes	Number	ZNF331 methylation status		*p* value
		Methylated	Unmethylated	
KRAS mutation				
Wide type	94	60	34	0.437
Mutation	42	30	12	
BRAF 600E mutation				
Wide type	133	87	46	1.000
Mutation	4	3	1	
CIMP				
CIMP-high	15	12	3	0.260
CIMP-low	121	77	44	
CACNA1G				
Methylated	29	22	7	0.271
Unmethylated	107	67	40	
IGF2				
Methylated	34	25	9	0.301
Unmethylated	102	64	38	
MLH1				
Methylated	21	13	8	0.804
Unmethylated	115	76	39	
RUNX3				
Methylated	14	11	3	0.379
Unmethylated	122	78	44

**Table 4:** Univariate and multivariate analysis of ZNF331 methylation status with disease-free survival (DFS) and overall survival (OS) in colorectal cancer patients

Clinical parameter	DFS				OS			
	Univariate analysis		Multivariate analysis		Univariate analysis		Multivariate analysis	
	HR (95% CI)	*p* value	HR (95% CI)	*p* value	HR (95% CI)	*p* value	HR (95% CI)	*p* value
Age (< 60 vs. ≥ 60 years)	1.099 (0.604–1.999)	0.757			1.132 (0.622–2.058)	0.685		
Gender (male vs female)	1.129 (0.603–2.114)	0.704			1.080 (0.577–2.023)	0.810		
ZNF331 (methylation vs. unmethylation)	0.273 (0.115–0.648)	0.003**	0.279 (0.117–0.664)	0.004**	0.261 (0.110–0.620)	0.002**	0.271 (0.114–0.646)	0.003**
Tumor differentiation (moderate or well vs. poor)	0.467 (0.254–0.857)	0.014*	0.727 (0.392–1.347)	0.311	0.471 (0.257–0.863)	0.015*	0.736 (0.397–01.365)	0.331
TNM stage (I–II vs. III–IV)	0.139 (0.062–0.312)	0.000***	0.151 (0.066–0.345)	0.000***	0.147 (0.065–0.332)	0.000***	0.164 (0.072–0.375)	0.000***
Tumor size (< 5 vs. ≥ 5 cm)	0.798 (0.438–1.453)	0.461			0.812 (0.446–1.478)	0.495		
Tumor location (left colon vs. right colon)	1.577 (0.810–3.072)	0.180			1.579 (0.811–3.075)	0.179		
KRAS mutation (wide type vs. mutation)	0.797 (0.418–1.520)	0.491			0.810 (0.425–1.545)	0.522		
BRAF 600E mutation (wide type vs. mutation)	0.488 (0.118–2.025)	0.323			0.396 (0.095–1.642)	0.202		
CIMP (high vs. low)	1.155 (0.412–3.242)	0.784			1.138 (0.405–3.192)	0.806		

*p < 0.05, **p < 0.01, ***p < 0.001

The expression of ZNF331 was evaluated by immunohistochemistry in 28 cases of available CRC and matched adjacent paraffin tissue samples (Fig. 2d). Reduced ZNF331 expression was found in 19 cases of CRC samples compared to adjacent tissue samples (Fig. 2e, *p* < 0.01). Among the 19 cases in which reduced expression of ZNF331 was detected, 16 cases were methylated, while in the 9 cases in which ZNF331 was expressed, only 3 cases were methylated. Reduced expression of ZNF331 was associated with promoter region methylation (*p* < 0.01, Fig. 2f). These results further suggest that the expression of ZNF331 is regulated by promoter region methylation in human CRC.

To further validate the methylation status and regulation of ZNF331 in human colorectal cancer, ZNF331 mRNA expression and promoter region methylation data were extracted from Genotype-Tissue Expression (GTEx) databases and the Cancer Genome Atlas (TCGA) (http://xena.ucsc.edu/). ZNF331 expression was detected by RNA sequencing (RNA-Seq) in 383 cases of CRC samples and 359 cases of normal colorectal mucosa. The levels of ZNF331 expression were significantly lower in CRC samples compared to normal colorectal mucosa samples (*p* < 0.001, Fig. 2g). Methylation of ZNF331 was analyzed by Illumina Infinium Human Methylation 450 (HM450) based on the methylation status of 14 CpGs in the promoter region. Methylation levels of ZNF331 were increased in CRC samples compared to adjacent tissue samples according to available data from 356 cases of CRC and 21 cases of matched adjacent tissue samples (*p* < 0.001, Fig. 2h). In the 356 cases of CRC samples, reduced expression of ZNF331 was associated with promoter region hypermethylation (*p* < 0.0001, Fig. 2i, j). These data further support our results in this study.

### ZNF331 suppresses cell proliferation in CRC cells

**Fig. 3:**
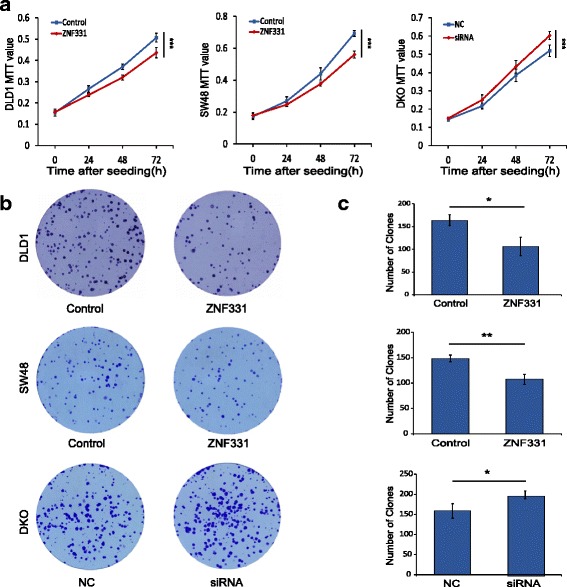
ZNF331 suppresses colorectal cancer cell proliferation. **a** The effects of ZNF331 on cell proliferation was measured by the MTT assay for 72 h in DLD1 and SW48 cell lines before and after restoration of ZNF331 expression and in DKO cells before and after knockdown of ZNF331 (***p < 0.001). **b**, **c** The effects of ZNF331 on colony formation in DLD1 and SW48 cell lines before and after restoration of ZNF331 expression and in DKO cells before and after knockdown of ZNF331 (*p < 0.05, **p < 0.01)

To evaluate the effects of ZNF331 on cell proliferation, cell viability was detected by MTT assays. The OD value were 0.507 ± 0.020 vs. 0.436 ± 0.023 in DLD1 and 0.693 ± 0.018 vs. 0.560 ± 0.021 in SW48 before and after restoration of ZNF331 expression (Fig. 3a). Cell viability was significantly reduced after restoration of ZNF331 expression in DLD1 and SW48 cells (both *p* < 0.001). The OD values were 0.520 ± 0.031 vs. 0.603 ± 0.022 (*p* < 0.001) before and after knockdown of ZNF331 in DKO cells (Fig. 3a). Cell viability was increased after knockdown of ZNF33 in DKO cells.

Colony formation assays were performed to evaluate the effect of ZNF331 on clonogenicity. The clone numbers were 164 ± 12.1 vs. 106 ± 20.3 in DLD1 cells and 148.7 ± 6.5 vs. 107.7 ± 9.9 in SW48 cells before and after restoration of ZNF331 expression. The number of clones was reduced after re-expression of ZNF331 in DLD1 and SW48 cells (both *p* < 0.01). The effect of ZNF331 on clonogenicity was further validated by knockdown of ZNF331 in DKO cells. The clone numbers were 158.7 ± 18.0 vs. 198.7 ± 10.0 before and after knockdown of ZNF331 in DKO cells (*p* < 0.05, Fig. 3b, c).

**Fig. 4:**
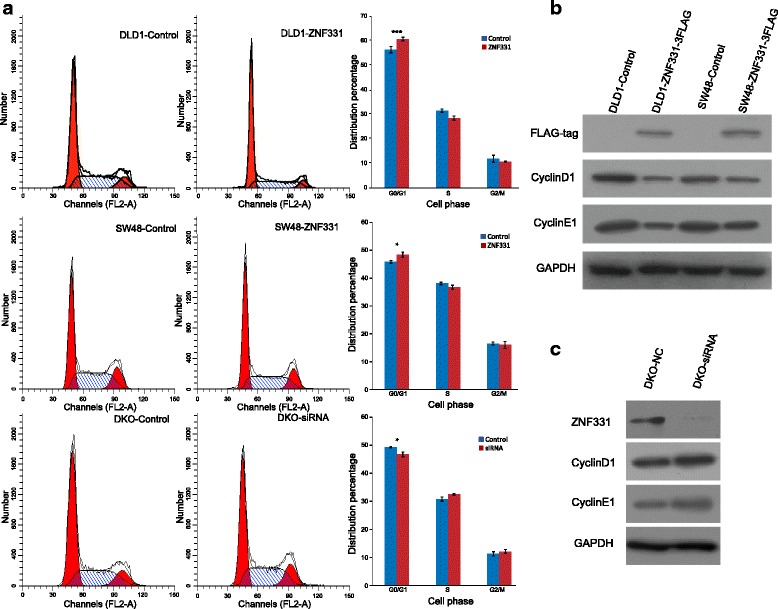
ZNF331 induces G0/G1 phase arrest in CRC cells. **a** Cell phase distribution in ZNF331 unexpressed and re-expressed DLD1 and SW48 cells and before and after siRNA knockdown of ZNF331 in DKO cells (*p < 0.05, ***p < 0.001.). **b** ZNF331-3FLAG, cyclin D1, and cyclin E1 were detected by Western blot in ZNF331 unexpressed and re-expressed DLD1 and SW48 cells. **c** Cyclin D1 and cyclin E1 were detected by Western blot in DKO cells before and after siRNA knockdown of ZNF331

The effects of ZNF331 on cell cycle were analyzed by flow cytometry. The cell phase distributions were 41.71 ± 0.32% vs. 44.22 ± 0.34% in G0/G1 phase, 41.35 ± 0.75% vs. 43.22 ± 0.83% in S phase, and 16.94 ± 0.53% vs. 12.55 ± 1.17% in G2/M phase before and after re-expression of ZNF331 in DLD1 cells. In the SW48 cells, the cell phase distributions were 45.53 ± 0.46% vs. 48.09 ± 0.93% in G0/G1 phase, 37.83 ± 0.44% vs. 36.43 ± 0.71% in S phase, and 16.31 ± 0.53% vs. 15.81 ± 1.29% in G2/M phase before and after re-expression of ZNF331. G1/S phase arrest was induced by restoration of ZNF331 in DLD1 and SW48 cells (*p* < 0.001, *p* < 0.05, Fig. 4a). To further validate these results, siRNA knockdown technique was employed. The cell phase distributions before and after knockdown of ZNF331 were as follows: G0/G1 phase 54.04 ± 0.24% vs. 51.25 ± 0.93%, S phase 33.71 ± 0.83% vs. 35.56 ± 0.32%, and G2/M phase 12.25 ± 0.82% vs. 13.19 ± 0.73%. G1/S arrest was induced by ZNF331 in DKO cells (*p* < 0.05, Fig. 4a). To further validate the effects of ZNF331 on cell cycle, the levels of cyclin D1 and cyclin E1 were examined by Western blot. The expression levels of cyclin D1 and cyclin E1 were reduced after re-expression of ZNF331 in DLD1 and SW48 cells, while the levels of cyclin D1 and cyclin E1 expression were increased after knockdown of ZNF331 in DKO (Fig. 4b, c). Taken together, these results suggest that ZNF331 inhibits cell proliferation in CRC.

### ZNF331 suppresses CRC cell xenograft growth in mice

**Fig. 5:**
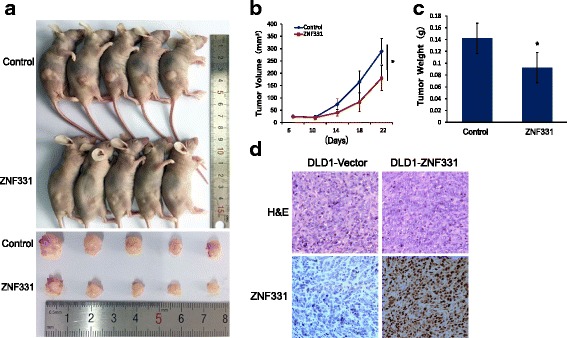
ZNF331 suppresses DLD1 cell tumor growth in colorectal cancer xenograft mice. **a** Results of ZNF331 re-expressed and unexpressed DLD1 cell xenografts in mice. Bottom: ZNF331 re-expressed colorectal cancer cells group; Top: control group. **b**, **c** Tumor growth curves and average weights of ZNF331 re-expressed and unexpressed DLD1 cell xenografts(*p < 0.05). **d** HE staining and IHC shows the expression of ZNF331 in ZNF331 re-expressed and unexpressed DLD1 cell xenografts (×200)

To further explore the role of ZNF331 in CRC, a xenograft mouse model was employed (Fig. 5a). ZNF331 unexpressed and re-expressed DLD1 cells were inoculated into the nude mice subcutaneously (Fig. 5d). The tumor volumes were 289.03 ± 52.22 mm^3^ in ZNF331 unexpressed DLD1 cell xenografts and 180.6 ± 50.28 mm^3^ in ZNF331 re-expressed DLD1 cell xenografts. The tumor volumes were smaller in ZNF331 re-expressed DLD1 cell xenograft mice compared to ZNF331 unexpressed DLD1 cell xenograft mice (*p* < 0.05, Fig. 5b). The tumor weights were 0.14 ± 0.03 g vs 0.09 ± 0.02 g in ZNF331 unexpressed and re-expressed DLD1 cell xenografts. The tumor weights were significantly different between these groups (*p* < 0.05, Fig. 5c). These results further suggest that ZNF331 suppresses CRC cell growth.

## Discussion

Classical zinc finger proteins (ZNFs) form the largest family of sequence-specific DNA-binding proteins and are encoded by 2% of human genes [20, 21]. Different types of zinc finger motifs influence a great diversity of biological functions, including differentiation, development, metabolism, apoptosis, autophagy, and stemness maintenance [22]. In addition to DNA binding, zinc finger motifs may interact with RNA, protein, and lipids [23, 24]. Thus, ZNFs may play more extensive roles in gene regulation.

As a member of this family, ZNF331 may serve as a transcriptional repressor [25]. ZNF331 was previously reported to suppress esophageal and gastric cancer growth [15, 26]. In this study, we found that ZNF331 is frequently methylated in human colorectal cancer and the expression of ZNF331 is regulated by promoter region methylation. Our results were supported by TCGA data. Methylation of ZNF331 is a potential colorectal cancer detection marker.

The CpG island methylator phenotype (CIMP) was first identified by Toyota et al. and has been extensively studied in colorectal cancer [27]. A cause or molecular mechanism for CIMP in colorectal cancer has not yet been identified. CIMP has been associated with environmental and lifestyle factors [28, 29], while there is no universal standard or consensus with respect to defining CIMP [30]. Weisenberger and colleagues identified a robust five-gene panel that recognized a distinct, heavily methylated subset of colorectal tumors that were also characterized by the *BRAF* mutation and MSI [31]. By screening eight methylation markers, Ogino et al. identified a four-gene methylation marker panel, including RUNX3, CACNA1G, IGF2, and MLH1, as a CIMP-high panel [17]. To explore the relationship of ZNF331 methylation and CIMP, we detected the methylation status of RUNX3, CACNA1G, IGF2, and MLH1, as well as KRAS or BRAF mutations in our cohort. No association was found between ZNF331 methylation and KRAS or BRAF mutations. No association was found between ZNF331 methylation and RUNX3, CACNA1G, IGF2, and/or MLH1 methylation. Our further studies indicate that methylation of ZNF331 is significantly associated with poor 5-year OS and 5-year DFS in CRC patients. Cox proportional hazards model analysis demonstrates that methylation of ZNF331 is an independent prognostic factor for poor 5-year OS and 5-year DFS in CRC. ZNF331 suppressed colony formation, cell proliferation, and induced G1/S arrest in colorectal cancer cells. ZNF331 suppressed human colorectal cancer cell tumor growth in xenograft mice. These results suggest that ZNF331 is a potential tumor suppressor in human CRC.

## Conclusions

ZNF331 is frequently methylated in human colorectal cancer, and the expression of ZNF331 is regulated by promoter region methylation. Methylation of ZNF331 is a poor prognostic marker in human colorectal cancer. ZNF331 may serve as a tumor suppressor in human colorectal cancer.

## Abbreviations

BSSQ: Bisulfite sequencing
CIMP: CpG island methylator phenotype
CRC: Colorectal cancer
DAC: 5-Aza-2′-deoxycytidine
DFS: Disease-free survival
ESCC: Esophageal squamous cell cancer
GTEx: Genotype-Tissue Expression
HM450: Illumina Infinium Human Methylation 450
IHC: Immunohistochemistry
LOH: Loss of heterozygosity
MSP: Methylation specific PCR
OS: Overall survival
PVDF: Polyvinylidene fluoride
RITA: Rearranged in thyroid adenoma
RPMI: Roswell Park Memorial Institute
RT-PCR: Reverse transcription PCR
TCGA: The Cancer Genome Atlas
TSS: Transcription start sites
ZNF331: Zinc finger protein
