# Correction to: Methylation of ZNF331 is an independent prognostic marker of colorectal cancer and promotes colorectal cancer growth

**DOI:** 10.1186/s13148-018-0467-2

**Published:** 2018-03-14

**Authors:** Yuzhu Wang, Tao He, James G. Herman, Enqiang Linghu, Yunsheng Yang, François Fuks, Fuyou Zhou, Linjie Song, Mingzhou Guo

**Affiliations:** 1 Department of Gastroenterology & Hepatology, Chinese PLA General Hospital, 28 Fuxing Road, Beijing, 100853 China; 2 Department of Geriatric Digestive System, Chinese PLA Navy General Hospital, 6 Fucheng Road, Beijing, 100048 China; 3 The Hillman Cancer Center, University of Pittsburgh Cancer Institute, Pittsburgh, PA 15213 USA; 4 Laboratory of Cancer Epigenetics, Free University of Brussels (U.L.B.), 1070 Brussels, Belgium; 5 Department of Thoracic Surgery, Anyang Tumor Hospital, Anyang, 455000 China; 6 Department of General Surgery, Chinese PLA General Hospital, 28 Fuxing Road, Beijing, 100853 China; 7 Medical College of NanKai University, Tianjin, 300071 China

## Correction

After publication of the original article [1], it came to the authors’ attention that a reference was omitted from the Background. The reference should have been inserted as [16] and is the following:

**Vedeld HM, Andresen K, Eilertsen IA, Nesbakken A, Seruca R, Gladhaug IP, Thiis-Evensen E, Rognum TO, Boberg KM, Lind GE: The novel colorectal cancer biomarkers CDO1, ZSCAN18 and ZNF331 are frequently methylated across gastrointestinal cancers. Int J Cancer 2015, 136:844–853.**

The last paragraph of the Background should have read as follows:

“Zinc finger protein 331 (ZNF331) was first identified from thyroid tumors [12]. It is also known as RITA (rearranged in thyroid adenoma), ZNF361, and ZNF463 [13]. The ZNF331 gene is located at chromosome 19q13.42, a region in which loss of heterozygosity (LOH) was detected in prostate cancer [14]. In our previous study, we found that the ZNF331 gene is frequently methylated in human esophageal squamous cell cancer (ESCC) and it serves as a tumor suppressor in ESCC [15]. **It was reported that the high methylation frequency of ZNF331 was found in some types of gastrointestinal cancer including CRC [16]. But the** function of ZNF331 in human CRC remains unclear. In this study, we analyzed the epigenetic regulation and the function of ZNF331 in human CRC.”

## References

[1] Wang (2017). Methylation of ZNF331 is an independent prognostic marker of colorectal cancer and promotes colorectal cancer growth. Clin Epigenetics.

